# Supplementary figures and images for: Range-wide phylogenomics of the Great Horned Owl (Bubo virginianus) reveals deep north-south divergence in northern Peru
Source: PeerJ. 2023 Aug 9;11:e15787. doi: 10.7717/peerj.15787 (PMC10422955; doi:10.7717/peerj.15787)

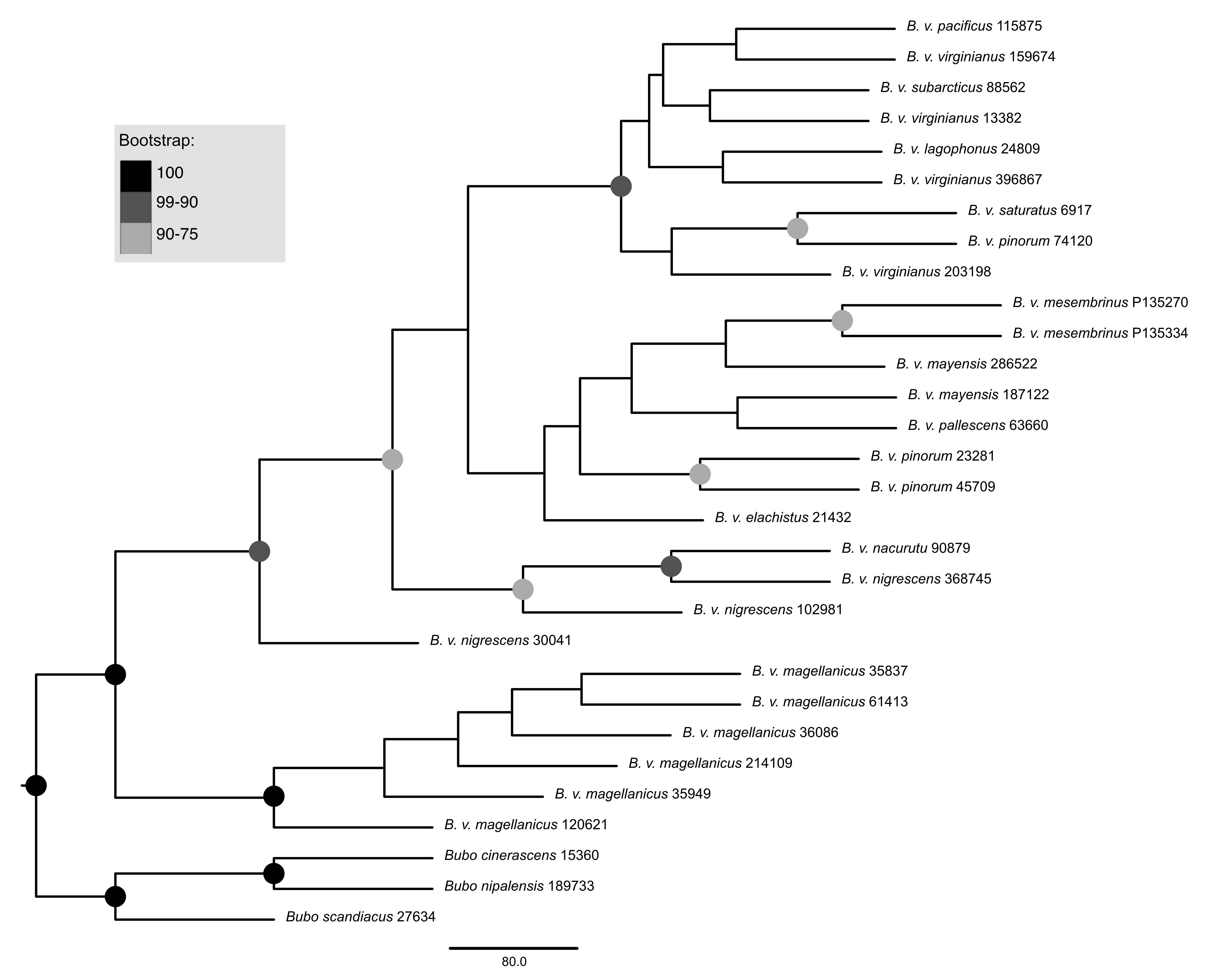

Supplement: Figure S1 — Support values are shown in the shaded circles on nodes. All nodes without support values are not highly supported. [file peerj-11-15787-s001.jpg]

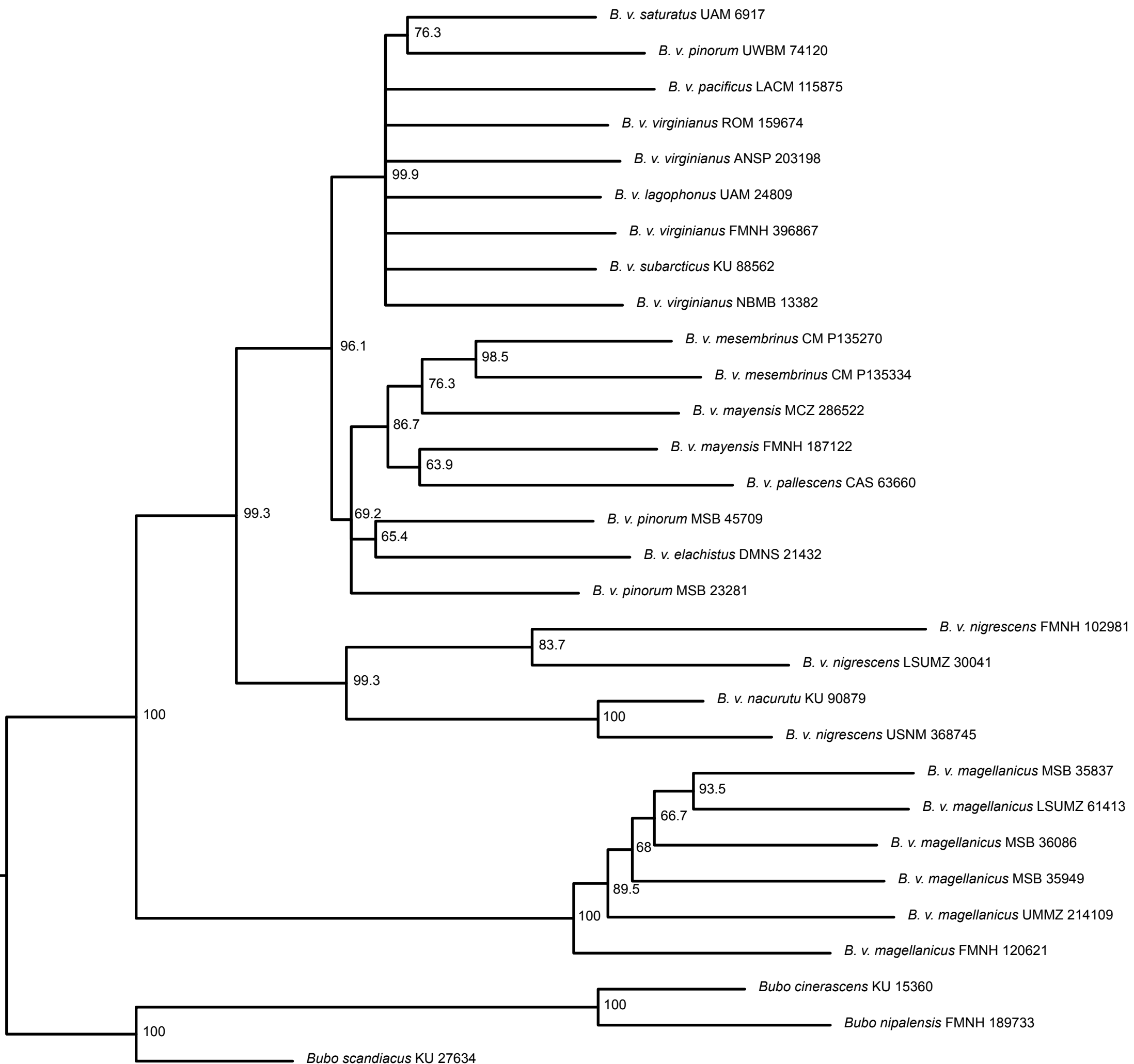

6.0E-5

Supplement: Figure S2 — Bootstrap values are listed to the right of nodes. Topologies with less than 50% bootstrap support are collapsed. [file peerj-11-15787-s002.pdf]

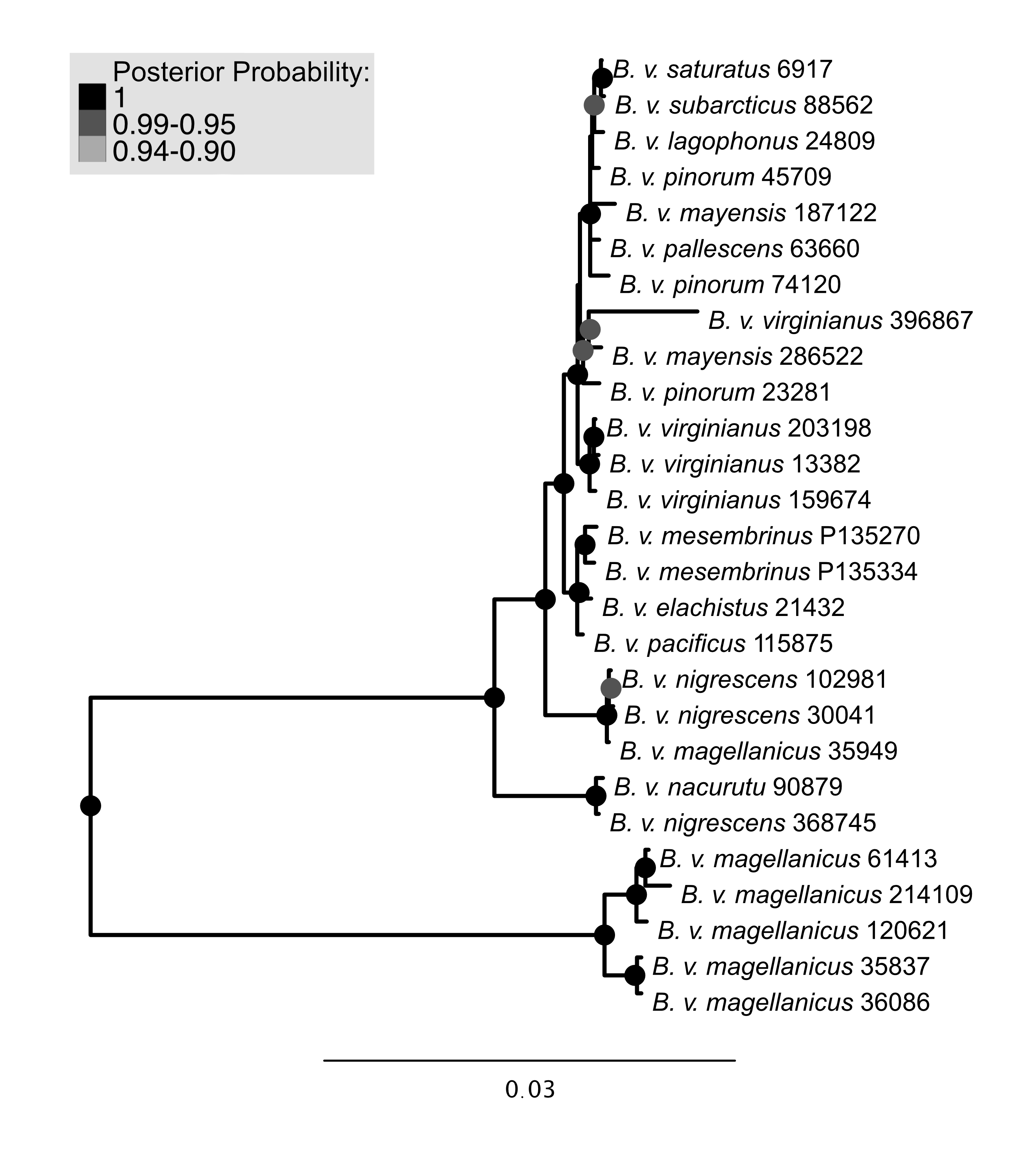

Supplement: Figure S3 — Support values shown in the shaded circles on nodes. Nodes without a circle have the lowest category of support. [file peerj-11-15787-s003.jpg]

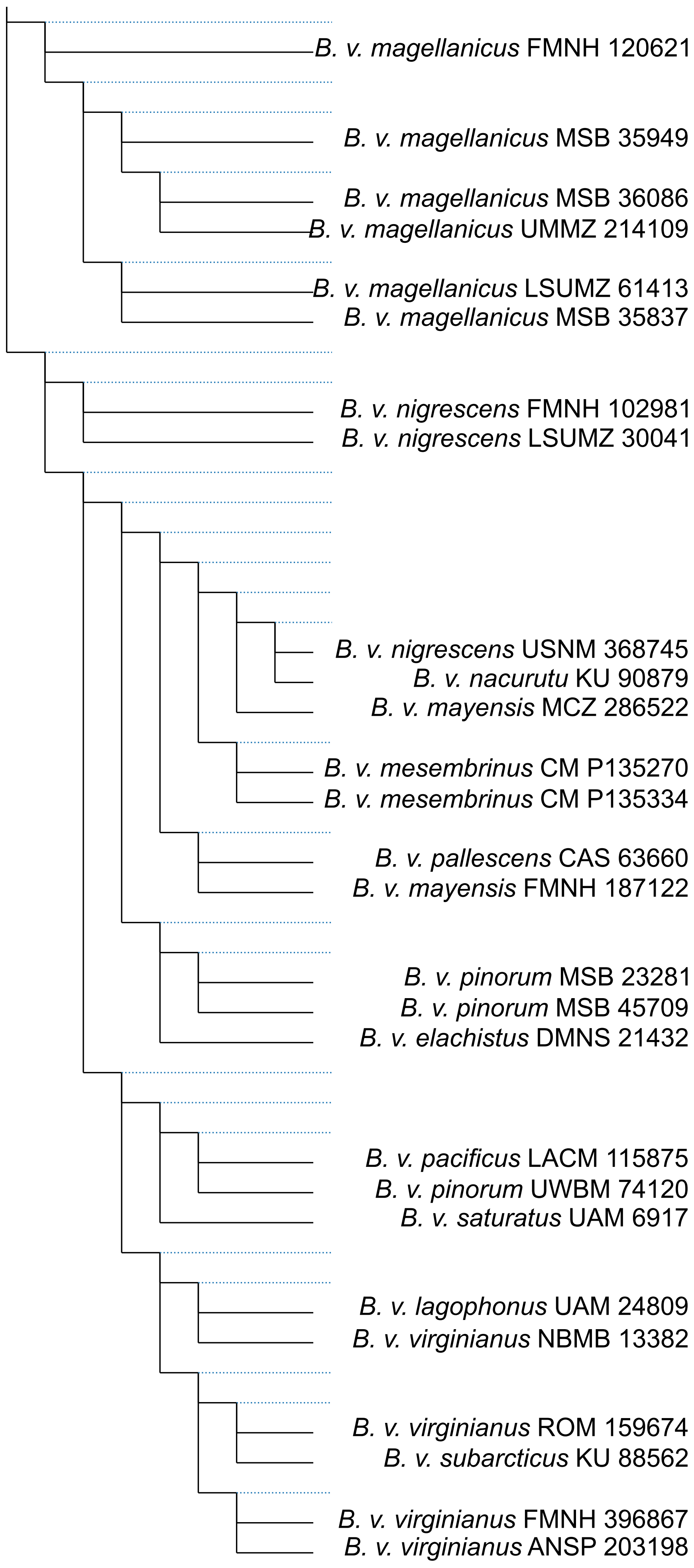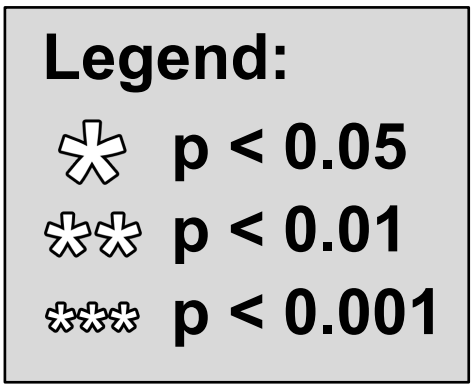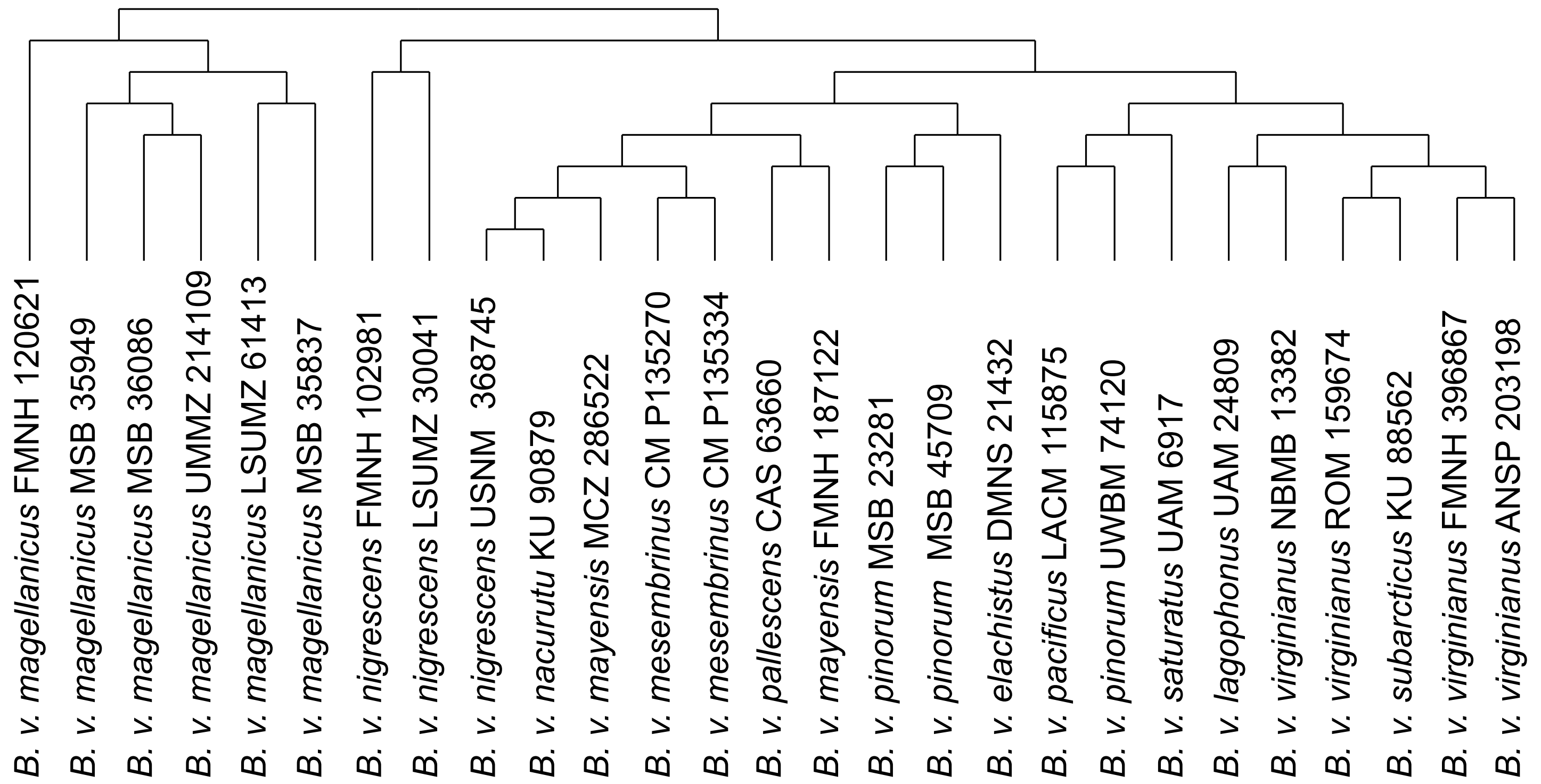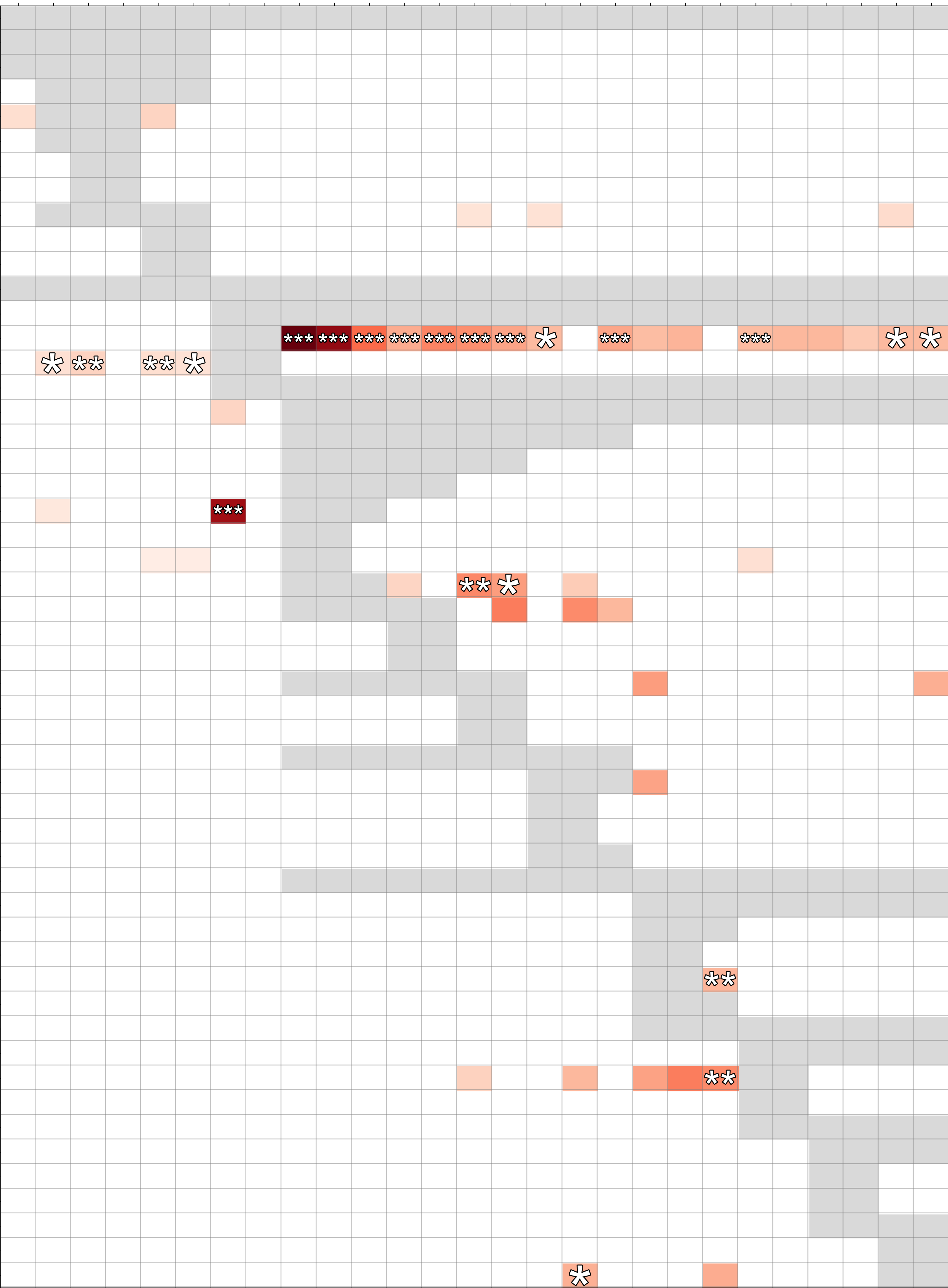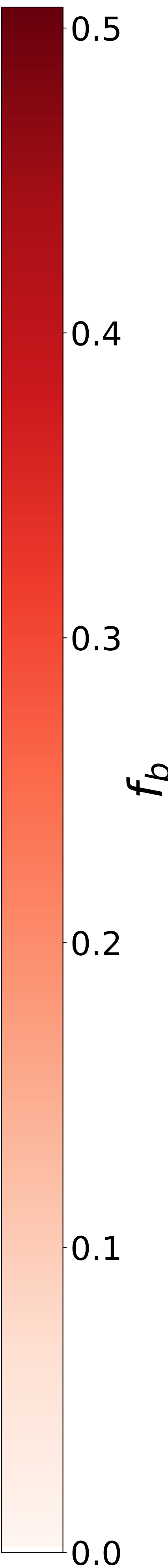

Supplement: Figure S4 — F-branch values are shown in varying shades of red. P-values are shown according to levels of significance: *** p < 0.001, ** p < 0.01, * p < 0.05. [file peerj-11-15787-s004.pdf]
